# A new urease-inhibiting formulation decreases ammonia volatilization and improves maize nitrogen utilization in North China Plain

**DOI:** 10.1038/srep43853

**Published:** 2017-03-08

**Authors:** Qianqian Li, Xiaoqing Cui, Xuejun Liu, Marco Roelcke, Gregor Pasda, Wolfram Zerulla, Alexander H. Wissemeier, Xinping Chen, Keith Goulding, Fusuo Zhang

**Affiliations:** 1 College of Resources and Environmental Sciences, Center for Resources, Environment and Food Security, Key Lab of Plant-Soil Interaction of MOE, China Agricultural University, Beijing 100193, China; 2 Institute of Geoecology, Technische Universität Braunschweig, 38106 Braunschweig, Germany; 3 German-Chinese Agricultural Center (DCZ), 55 Nongzhan Beilu, Chaoyang District, Beijing 100125, China; 4 BASF SE, Agricultural Center Limburgerhof, Speyerer Strasse 2, 67117 Limburgerhof, Germany; 5 The Sustainable Soils and Grassland Systems Department, Rothamsted Research, Harpenden AL5 2JQ, UK

## Abstract

Overuse of urea, low nitrogen (N) utilization, and large N losses are common in maize production in North China Plain (NCP). To solve these problems, we conducted two field experiments at Shangzhuang and Quzhou in NCP to test the ability of a newly developed urease inhibitor product Limus^®^ to decrease NH_3_ volatilization from urea applied to maize. Grain yield, apparent N recovery efficiency (RE_N_) and N balance when using urea applied with or without Limus were also measured over two maize growing seasons. Cumulative NH_3_ loss in the two weeks following urea application without Limus ranged from 9–108 kg N ha^−1^, while Limus addition significantly decreased NH_3_ loss by a mean of 84%. Urea with Limus did not significantly increase maize yields (*P* < 0.05) compared with urea alone. However, a significant 11–17% improvement in RE_N_ with Limus was observed at QZ. The use of urea-N plus Limus would permit a reduction in N applications of 55–60% compared to farmers’ practice and/or further 20% N saving compared with optimized urea-N rate (150 kg N ha^−1^, based on N requirement by target yield of 7.5 t ha^−1^), and would achieve the same maize yields but with significantly decreased NH_3_ loss and increased N utilization.

Ammonia (NH_3_) is one of the key reactive N (Nr) components and the major alkaline gas in the atmosphere. NH_3_ directly and/or indirectly contributes to the eutrophication of aquatic ecosystems, soil acidification when nitrified and leached, and the decline in plant biodiversity through its deposition; it also damages human health through secondary aerosol or particle formation (e.g., PM_2.5_)^1^,^2^,^3^. In China, the total NH_3_ emission was estimated to be 9.8–20.4 Tg per year in the 2000s, 33–46% of which was derived from N fertilizer application^4^,^5^. Another study^6^ showed that the NH_3_ loss was up to 3.7 Tg in the North China Plain (NCP) in 2004, of which N fertilizer contributed 54% (and the rest was mainly from animal manures).

Urea accounts for around 60% of total N fertilizer consumption in China, but it has a large potential for NH_3_ loss, amounting to more than 20% in arable soils with a pH > 7 (e.g. those in the NCP)^7^,^8^,^9^. Strategies to decrease NH_3_ loss from urea-N application to croplands have included the incorporation of surface applied N, deep placement of N fertilizer, and irrigation immediately following N topdressing^10^,^11^. However, on smallholder farms, these conservation practices are frequently not used due to labour shortages and delayed irrigation, so NH_3_ loss is expected to be high. These mitigation practices are even more difficult to apply if the N is applied as a top-dressing or as split applications at selected crop growth stages.

In addition, because of the long-term pressure for food security in China, farmers tend to apply excessive amounts of N fertilizer as an “insurance” to meet crop needs. In the NCP, N recovery efficiency (RE_N_) from urea applied according to farmers’ practice averaged only 15% across 148 on-farm maize experiments^12^ and 10–19% in a two-year maize based cropping experiment^13^, suggesting a large opportunity for reducing N losses and improving RE_N_ in current urea-N management practices without sacrificing maize yields^14^.

There is, therefore, a strong demand for options to decrease NH_3_ losses while achieving higher RE_N_ and crop yields. Application of a urease inhibitor could contribute to achieving these objectives, as it should delay urea hydrolysis, decrease NH_3_ losses and improve N utilization^15^. A new urease inhibitor product Limus^®^, as a mixture of 75% N-(n-butyl) thiophosphoric triamide (NBPT) and 25% N-(n-propyl) thiophosphoric triamide (NPPT), was recently developed by BASF SE (a German Company). A laboratory study proved that the mixture of NBPT and NPPT (e.g. 75% vs. 25% or 50% vs. 50%) could achieve better effects in decreasing NH_3_ loss than single urease inhibitor (NBPT or NPPT)^16^. Limus product (here referring to plain urea amended with Limus) can be broadcasted on soil surface without extra irrigation or soil incorporation. Recently our previous study demonstrated the positive effects of Limus on decreasing NH_3_ loss in winter wheat at three locations and improving yield and N utilization with 55% less famer’s N practice under once application in wheat at one of three locations in north and northwest China^17^. However, the effects of Limus on maize, the largest crop in NCP, were not evaluated under field trials. Considering completely different meteorological (e.g. warm and humid in maize vs. cold and dry in wheat) and soil (e.g. different urease activities) conditions, we hypothesize that the effects of Limus on urease inhibition and NH_3_ loss mitigation may be lower in maize season than in wheat season. This is because the warm and humid condition will accelerate urea’s hydrolysis and the degradation of Limus.

To systematically evaluate the role of Limus in reducing NH_3_ volatilization and improving RE_N_ and grain yields in maize season, we conducted two typical field experiments over two years at Shangzhuang (SZ) and Quzhou (QZ). We wanted to (1) test our hypothesis (e.g. if Limus shows lower role in mitigating NH_3_ loss in maize than in wheat) and (2) develop a practical recommendation to minimize NH_3_ loss and achieve high yields and N utilization in maize in NCP.

## Results

### Cumulative NH_3_ losses

After each N application over the two-year trial at each site, NH_3_ volatilization from urea alone at SZ ranged from 9 to 31 kg N ha^−1^ (accounting for 4–38% of N application) compared to 0 to 2 kg N ha^−1^ (accounting for 0–2% of N application) from urea amended with Limus, while at QZ NH_3_ loss ranged from 18 to 59 kg N ha^−1^ (accounting for 18–61% of N application) for urea alone compared to 1 to 17 kg N ha^−1^ (accounting for 1–28% of N application) from urea with Limus (Table 2). Compared with N_con_, N_opt_ significantly (*P* < 0.05) decreased total NH_3_ loss by up to 2–9 kg N ha^−1^ at SZ and 13–23 kg N ha^−1^ at QZ, while urea amended with Limus significantly decreased NH_3_ loss by 70–100% at the V3, and 63–100% at the V12 growth stage compared to N_opt_ at both sites. In contrast, no significant differences in NH_3_ volatilization were observed between the various Limus treatments (Table 1) at SZ in both years and at QZ in the first year. A significant reduction of total NH_3_ loss by 14 kg N ha^−1^ (*P* < 0.05) was observed in N_80%opt/L-1_ compared to N_opt/L_ and N_80%opt/L_ at QZ in the second year.

At SZ, total cumulative NH_3_ loss varied from 0 to 37 kg N ha^−1^ (accounting for 0–19% of urea-N) under the different fertilizer treatments, while NH_3_ loss significantly decreased to almost zero kg N ha^−1^ (78–100% reduction, *P* < 0.05) when Limus was added. At QZ, Limus addition reduced total NH_3_ loss by 38–55 kg N ha^−1^ (*P* < 0.05), corresponding to a 65–90% decrease in NH_3_ loss (Table 2). The greatest decrease in total NH_3_ losses due to Limus addition occurred in the second year at QZ. At SZ, even though only 5–11 kg N ha^−1^ NH_3_ was emitted at V12 in the urea-only treatments, urea amended with Limus decreased NH_3_ volatilization by 5–7 kg N ha^−1^ (*P* < 0.05), corresponding to a 78–100% reduction of NH_3_ loss.

### Time courses of NH_3_ fluxes

**Figure 1:**
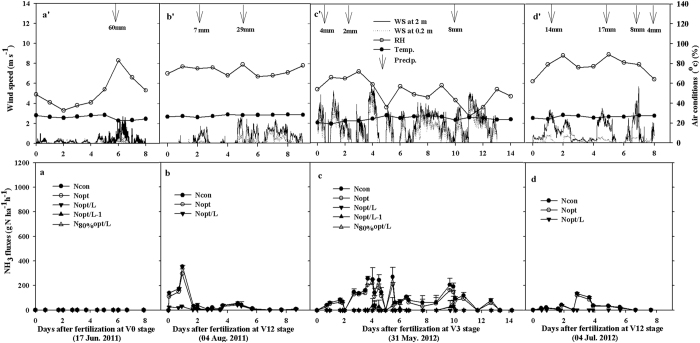
NH_3_ fluxes measured by a calibrated Dräger-Tube Method (DTM) following urea (±Limus) application at V0 or V3 (seeding or three-leaf extension) and V12 (12-leaf extension) growth stages of summer maize in 2011 and 2012 at Shangzhuang (SZ, Fig. 1a–d) and corresponding time courses of precipitation (Precip., mm), wind speed (WS, 2.0 m and 0.2 m heights), air temperature (Temp., °C) and relative humidity (HM, %) (Fig. 1a’–d’). Bars denote standard deviations of NH_3_ fluxes on four replicate plots.

**Figure 2:**
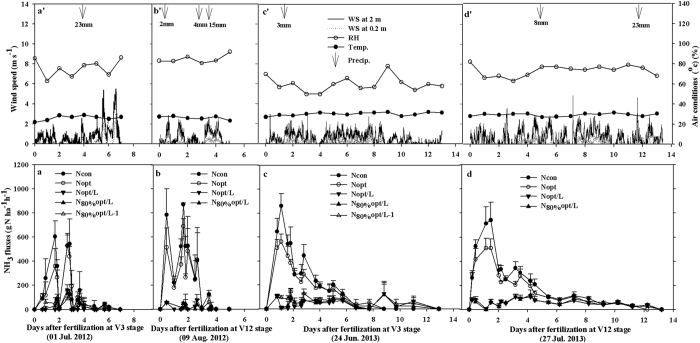
NH_3_ fluxes measured by a calibrated Dräger-Tube Method (DTM) following urea (±Limus) application at V3 (three-leaf extension) and V12 (12-leaf extension) growth stages of summer maize in 2012 and 2013 at Quzhou (QZ, Fig. 2a–d) and corresponding time courses of precipitation (Prec., mm), wind speed (WS, 2.0 m and 0.2 m heights), air temperature (Temp., °C) and relative humidity (RH, %) (Fig. 2a’–d’). Bars denote standard deviations of NH_3_ fluxes on four replicate plots.

The dynamics of NH_3_ fluxes and weather conditions (precipitation, air temperature, relative humidity and wind speed at two heights, etc.) in the two study years at SZ and QZ are shown in Figs 1 and 2, respectively. Precipitation events occurred frequently, while the relative humidity ranged from 35–90% (mostly above 55%), and the air temperature mainly around 22–33 °C during the different periods of NH_3_ volatilization at SZ and QZ (Figs 1a’–d’ and 2a’–d’). The daily NH_3_ emission rates in the two N fertilization events (at V0 or V3 and V12 growth stages) varied between the two study years at SZ (Fig. 1a–d); no NH_3_ volatilization was detected at V0 in June 2011 (Fig. 1a). At V12 in August 2011 (Fig. 1b), the daily volatilization losses were higher in N_con_ and N_opt_, in which volatilization peaked at 360 and 300 g N ha^−1^ h^−1^, respectively, on the first day after surface application of urea. In contrast, urea with Limus (N_opt/L_) lost a daily maximum of only about 30 g N ha^−1^ h^−1^ on the first day after surface application. The NH_3_ fluxes decreased to 0–50 g N ha^−1^ h^−1^ on all of the treatments two days later.

Compared with the high NH_3_ fluxes at SZ after N fertilization at V12 in 2011, maximum volatilization rates were observed with urea alone at V3 in 2012. The highest NH_3_ fluxes from N_con_ and N_opt_ at SZ occurred from the 3^rd^ to the 10^th^ day after fertilization, while much lower peak fluxes (0–30 g N ha^−1^ h^−1^) from the Limus treatments (N_opt/L_, N_opt/L-1_, N_80%opt/L-1_) were measured on the 4^th^ and 10^th^ days after fertilization at V3 in 2012 (Fig. 1c).

At QZ, the daily NH_3_ volatilization rates (Fig. 2a–d) were approx. 3 times higher than those at SZ (Fig. 1a–d) over the two-years. Fluxes at QZ were higher in the N_con_ and N_opt_ treatments, peaking at 400–800 and 200–700 g N ha^−1^ h^−1^, respectively, during the first 3 days after urea application. Thereafter, they quickly decreased to 0–200 g N ha^−1^ h^−1^ for both N_con_ and N_opt_ (Fig. 2a–d). In contrast, NH_3_ fluxes in the urea with Limus treatments (N_opt/L,_ N_80%opt/L,_ N_80%opt/L-1_) were only 0–150 g N ha^−1^ h^−1^ after application. Generally, similar maximum NH_3_ fluxes (600–800 g N ha^−1^ h^−1^) at QZ were measured at each fertilization event in spite of slightly different patterns of flux. Following N fertilization at V3 and V12, NH_3_ volatilization was only detected for 5–7 days during the first year (Fig. 2a–b), while the volatilization period lasted longer (up to 12 days) in the second year (Fig. 2b–c).

### Grain yield and N recovery efficiency

**Figure 3:**
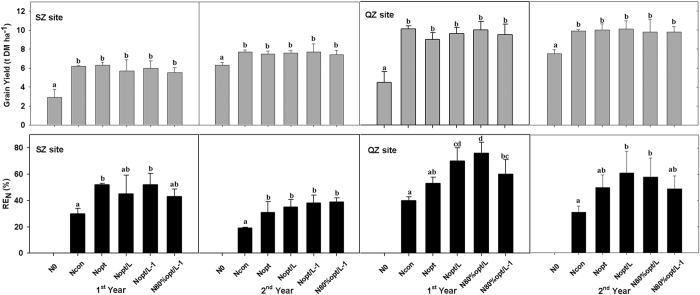
Maize grain yield and apparent nitrogen recovery efficiency (RE_N_ indicates the percentage of fertilizer N recovered in aboveground plant biomass) in the two years at Shangzhuang (SZ) and Quzhou (QZ). Different letters above the bars indicate the significance under *P* < 0.05.

Lower grain dry matter (DM) yields (2.9–6.3 t DM ha^−1^ with no N) were found at SZ compared with those at QZ (4.5–7.5 t DM ha^−1^ with no N) (Fig. 3). At SZ, the grain yields ranged from 5.5 to 7.7 t DM ha^−1^ during the first and second years under different N application treatments, while the grain yields ranged from 9.0 to 10.1 t DM ha^−1^ d at QZ site. Totally, N fertilizer application significantly (p < 0.05) improved maize grain yield by 93–135% (1^st^ year) and 21–33% (2^nd^ year) but no statistically significant differences (*P* > 0.05) were observed between N treatments. Even the most efficient (in terms of RE_N_) treatment N_80%opt/L_ obtained maize yields similar to the other N treatments at both sites.

The RE_N_ (N recovery efficiency, an indicator of N use efficiency) was between 30% (N_con_) and 52% (N_opt_ & N_opt/L-1_) in the first year, and 19% (N_con_) to 39% (N_80%opt/L-1_) in the second year at SZ (Fig. 3). In contrast, the RE_N_ was consistently higher (ranging from 1 to 26%) at QZ than at SZ across all N treatments during both years on average. The RE_N_ was lower in N_con_ than in the N_opt_ treatments because of the greater amount of N applied to N_con_ at both sites. No significant differences between optimized N fertilization (±Limus) were found at SZ, but the use of Limus (N_opt/L_) at QZ led to an average RE_N_ increase by 17% and 11% when compared to N_opt_ in the 1^st^ and 2^nd^ years, respectively. Also RE_N_ in the N_80%opt/L_ treatment was 23% (*P* < 0.05) higher than in N_opt_ in the first year at QZ but, on average across the two years and two sites, applying N_80%opt/L-1_ did not result in significant differences in RE_N_ (ranging from 39 to 60%) compared with N_opt_ (ranging from 31 to 53%).

### Nitrogen balance

Nitrogen balances are shown in Table 3. The “other N loss” in Table 3 comprises all N loss pathways other than NH_3_ volatilization (e.g., nitrification/denitrification, N leaching) as well as possible errors. Relatively low mineral N (N_min_) in the 0–1 m soil profiles before sowing was observed at both SZ (68 kg N ha^−1^) and QZ (35 kg N ha^−1^) in the first year, while relatively high N_min_ before sowing was measured on each plot at both SZ (62 to 220 kg N ha^−1^) and QZ (59 to 120 kg N ha^−1^) in the second year (Table 3). Apparent N mineralization based on an N balance in N_0_ was 33 kg N ha^−1^ at SZ, and 57 kg N ha^−1^ at QZ during two years on average.

At SZ, the residual soil N_min_ at harvest in N_con_ was 212 and 337 kg N ha^−1^ during the first and second years, respectively. These values were significantly higher than those in the optimized urea ± Limus treatments (72–162 kg N ha^−1^) during both years. The apparent N loss based on an N balance in N_con_ at SZ was 28 and 83 kg N ha^−1^ during the first and second years, but only 16–44 kg N ha^−1^ in the optimized urea ± Limus treatments (Table 3). At QZ, the highest residual N_min_ was measured in N_con_ (averaging 107 kg N ha^−1^), while the amounts were about 67% less in the N_opt_ treatment (averaging 34 kg N ha^−1^) and 56% lower in the urea with Limus (N_opt/L,_ N_opt/L-1,_ N_80%opt/L-1_) treatments (averaging 47 kg N ha^−1^). Likewise, the highest mean apparent N loss of 118 kg N ha^−1^ occurred in N_con_, followed by 75 kg N ha^−1^ in N_opt_, and 40 kg N ha^−1^ in N_opt/L,_ N_optL-1,_ and N_80%opt/L-1_. As a whole, both the highest residual soil N_min_ and apparent N losses were in N_con_ plots at both sites. Relatively low apparent N loss was observed for treatments with Limus compared to N_opt_. Moreover, the proportions of residual soil N_min_ to apparent N losses differed at SZ and QZ: residual soil N_min_ accounted for 80% of the N surplus (76 to 85%) at SZ but only 49% (40 to 58%) at QZ. Compared with the 1^st^ year, the N surplus under farmers’ practice (N_con_) increased by 87 (at QZ) and 180 (at SZ) kg N ha^−1^, while only by 45 (at QZ) and 69 (at SZ) kg N ha^−1^ with optimized N fertilization (N_opt,_ N_opt/L_) in the 2^nd^ year.

## Discussion

Our research confirmed that conventional urea application to maize caused large NH_3_ losses (average 10–36% of urea-N applied) in the NCP. Similar research by Su *et al*.^9^ found NH_3_ losses of up to 26–33% of surface-applied urea at the V3 and V10 growth stages of maize production in the NCP. Optimized urea amended with Limus significantly decreased NH_3_ losses by a mean of 84% and improved RE_N_ in some cases, consistent with our previous results in winter wheat (e.g. a mean of 83% in NH_3_ loss mitigation)^17^. Our results deny our prior hypothesis that Limus may have lower role in mitigating NH_3_ loss and improving RE_N_. We expect that the trade-offs between meteorological conditions (warm and humid in maize vs. cold and dry in wheat) and NH_3_ volatilization processes (high flux with short volatilization period in maize vs. low flux with long volatilization period in wheat) could explain why Limus effects on maize (the present study) are comparable to that on wheat^17^. In addition, the inhibition period of up to 14 days allowed sufficient time for rain to leach the urea into the subsoil and so prevent further NH_3_ losses (Figs 1 and 2).

The dynamics of NH_3_ fluxes were mostly determined by the weather (e.g., precipitation, air temperature and wind speed) after N application (Figs 1 and 2). At SZ, for example, NH_3_ loss was only detected during the V3 growth stage in 2012, when initial environmental conditions (2–4 mm light precipitation and 60–78% relative humidity) were favorable for NH_3_ volatilization. In contrast, the low relative humidity (35–50%) and soil moisture content, together with a heavy rainfall (60 mm) on the 6^th^ day after N top dressing during the V0 growth stage in 2011 were unfavorable for NH_3_ volatilization. The magnitude of NH_3_ losses was largely influenced by small temporal differences in the weather and initial soil moisture content^18^,^19^. Some farmers in China broadcast N fertilizer after precipitation. This would further increase NH_3_ volatilization risk because high air humidity or limited rainfall provide adequate moisture for urea hydrolysis and cause the desorption of ammoniacal N from the soil^20^,^21^. In contrast, heavy rainfall effectively mitigates NH_3_ losses by leaching unhydrolized urea into the soil profile where it will be retained^11^,^22^. This was clearly demonstrated during the two maize growing seasons at QZ (Fig. 2a–d). Here moist initial soil conditions favoured rapid urea hydrolysis and promoted the initial NH_3_ loss. However, the high NH_3_ fluxes then rapidly decreased and stopped after precipitation (15–23 mm). In the NCP, we find that NH_3_ volatilization will not be inhibited unless the daily precipitation is ≥7 mm (Figs 1a’–d’ and 2a’–d’).

Additionally, the NH_3_ flux from urea at QZ was about 3 times higher than that at SZ over the two seasons (Figs 1a–d). This can partly be explained by the much higher urease activity in the QZ soil (13.0 μg N g^−1^ soil h^−1^ or 29.3 kg N ha^−1^ h^−1^) than the SZ soil (4.2 μg N g^−1^ soil h^−1^ or 9.5 kg N ha^−1^ h^−1^). High soil urease activity was reported to have a close positive relationship with the timing of urea hydrolysis as well as NH_3_ loss^23^,^24^. Nevertheless at SZ, NH_3_ loss still amounted to 31% of the urea-N applied (75 kg N ha^−1^) during the V3 growth stage in 2012 (Table 2). This confirms that urea hydrolysis-induced NH_3_ volatilization is mostly dependent upon meteorological (e.g. amount of precipitation and wind speed) and soil (e.g. soil humidity and soil temperature) conditions^15^,^18^. In the current study, we found that the NH_3_ loss rates in maize seasons (2012/13) were nearly 20% higher than those in the previous winter wheat season (2011/12) at QZ with same amount of urea^17^. One factor that possibly influenced the different losses was air-soil temperature since the higher temperature in the summer maize growing season may have increased microbial activity and hydrolysis of urea fertilizers, also more NH_3_ in gaseous phase and less in solution phase at higher temperature^15^,^23^.

Considering the high potential for NH_3_ loss in the NCP, with most soils at pH > 7.5, we expected that Limus will be very effective in decreasing NH_3_ emissions in this region. Our two-year experimental data confirm this role of Limus at both SZ and QZ sites in NCP.

Some previous reports have shown that the use of a urease inhibitor could increase the yields of crops^25^,^26^, while others did not show any significant yield increases^27^,^28^,^29^. A meta-analysis conducted by Abalos *et al*.^30^ found that urease inhibitors had a positive effect on crop yield (10%) and N use efficiency (12%), but their effectiveness was dependent on environmental and management factors. Our study clearly demonstrates a significant effect of Limus on mitigating NH_3_ volatilization but no significant effect on yield. During our first study year (2011 at SZ and 2012 at QZ), the initial soil N_min_ contents were low (35–68 kg N ha^−1^) because of previous N depletion (Table 3). However, grain yields were maintained under treatments of urea with Limus (N_opt/L-1_ & N_80%opt/L-1_) applied only once at V0 or V3 (Fig. 3). High NH_3_ losses of 42–55 kg N ha^−1^ (accounting for 20–28% of total N applied) were measured at QZ during the first maize season (2012) in the absence of Limus but, unexpectedly, maize grain yield was not improved by using Limus despite initial soil N_min_ contents being less than half of those at SZ. However, the RE_N_ of urea with Limus (N_opt/L_ & N_80%opt/L_) significantly increased by about 20% and 33% compared to N_opt_ (53%) and N_con_ (40%), respectively, and there was an increase in maize N uptake. During the 2^nd^ study year, the sum of inputs to soil mineral N (including initial soil N_min_, N fertilizer, plus contributions from N mineralization) increased to a mean of 363 kg N ha^−1^ at SZ and 324 kg N ha^−1^ at QZ, respectively (Table 3). Therefore at both sites, despite the NH_3_ loss of 28–37 kg N ha^−1^ at SZ and 86–109 kg N ha^−1^ at QZ, there was still more than sufficient soil N_min_ available to meet crop requirements without the use of Limus. However, residual mineral N contents were approx. 30 kg N ha^−1^ higher (ranging from 11 to 52 kg N ha^−1^) in treatments with Limus (N_opt/L_), compared to N_opt_ treatments (Table 3), showing the N conserving effect of Limus and so the need to apply less N fertilizer to the following crop.

Research has shown that continually increasing the amount of N applied does not produce a sustained increase in crop productivity due to diminishing returns^31^. In our experiments, compared with the optimized urea-N treatments, grain yield was not significantly increased by the larger amounts that farmers applied (N_con_), while the RE_N_ of N_con_ was significantly reduced to 30% at SZ and 40% at QZ in the 1^st^ year and 19% at SZ and 31% at QZ in the 2^nd^ year. Previous observations indicated that RE_N_ in the NCP has decreased to <20% under current farming N practice^12^, because of excessive N fertilizer use by farmers, which is substantially less than the 30–35% achieved in the 1980 s in China^10^. As a consequence, the various N loss pathways including N leaching, NH_3_ volatilization, and N_2_O emission^5^,^13^ cause environmental pollution, such as enhanced N deposition^3^, pollution haze^32^, increased greenhouse gas emissions^33^, and soil acidification^34^. These problems have become increasingly serious in rapidly developing nations and their consequences are important on a global scale^1^,^35^. In addition, the excessive application of N fertilizer has been shown to lead to the accumulation of nitrate N in the soils of the NCP^12^. We found that the residual nitrate N retained in the 0–1 m soil profile after harvest ranged from 203 kg N ha^−1^ to 310 kg N ha^−1^ at SZ after the 2-year study. After harvesting maize, Ju *et al*.^36^ reported nitrate N to be 275 kg N ha^−1^ in the top 0.9 m soil layer and 213 kg N ha^−1^ in the 0.9–1.8 m soil layer in Shandong province in the NCP, which was a potential threat to shallow groundwater quality through nitrate N leaching. Over-application of urea-N is common in Chinese intensively managed croplands due to the low economic penalties associated with yield losses and the relatively low price of urea fertilizer^37^. The optimized urea treatment with or without Limus maintained yields and achieved higher RE_N_ values (51% with urea alone and 62% with urea with Limus at QZ, and 41% and 42% at SZ, respectively). This and the much smaller applications of N fertilizer (by about 48% compared to farmer’s practice) led to less Nr losses and a smaller residual soil N_min_. A further 20% reduction in optimized N rate by using urea plus Limus (N_80%opt/L-1_: 120 kg N ha^−1^, applied at V0 or V3) shows that Limus can maintain maize yields at a mean of approx. 6.5 t DM ha^−1^ at SZ and 9.6 t DM ha^−1^ at QZ while increasing the RE_N_ to an average of 41% at SZ and 55% at QZ, and at the same time saving labour. The once application practice of urea with Limus (e.g. N_80%opt/L-1_) should be consistent to Chinese smallholder farmers’ requirement: fertilization as simple as possible.

Although these practices represent a large step forward, increasing rather than merely maintaining crop yields remains a fundamental challenge. New research should quantify the economics (especially the environmental benefit of NH_3_ reduction) of using a urease inhibitor (e.g. Limus) across a range of soils, management, and climate variables.

## Methods

### Study sites

The North China Plain (NCP) is the main maize (and wheat) production region in China, accounting for 39% of total national maize production^13^. The climate conditions are sub-humid continental. Mean annual rainfall is 500–700 mm, with ~70% of precipitation occurring during the maize growing season. The field experiments were conducted for two consecutive years at two typical sites in the NCP: (1) Shangzhuang (SZ, 40°05′ N and 116°12′ E) in Beijing, during the 2011 and 2012 maize seasons; (2) Quzhou (QZ, 36°58′ N and 115°12′ E) in Hebei, during the 2012 and 2013 maize seasons. The soil types of SZ and QZ are fluvo-aquic soil and alluvial soil, respectively. Both soils are of calcareous loamy and silty texture and representative of the NCP. The initial soil pH at both (1:2.5 soil water ratio) was 7.9 ~ 8.0 and total N content 0.6 ~ 0.7 g kg^−1^. Urease activity was 4.2 μg N g^−1^ soil h^−1^ and 13.0 μg N g^−1^ soil h^−1^ before seeding in the SZ and QZ soils, respectively. And soil organic matter content was 9.7 and 12.0 g kg^−1^ in the SZ and QZ soils.

### Application of fertilizers and crop management practices

Before planting, phosphorus (calcium superphosphate, 60 kg P_2_O_5_ ha^−1^) and potassium (potassium sulphate, 60 kg K_2_O ha^−1^) fertilizers were applied to the plots, according to local mechanical tillage practices. Table 1 shows the detailed information on the N and Limus treatments at the sites, and there were six N treatments at each of the two maize experiments. All of the urea and urea plus Limus were surface applied in a randomized block design to four replicate plots of 30–40 m^2^. Five of the six treatments were the same at both sites: N_0_, no N fertilizer; N_con_, conventional urea (270–300 kg N ha^−1^); N_opt_, optimized urea (150 kg N ha^−1^); N_opt/L_, optimized urea amended with Limus (150 kg N ha^−1^), applied at two doses differently (SZ) or equally (QZ) at V0 (seeding, only at SZ in 2011) or V3 (three-expanded leaves) and V12 (twelve-expanded leaves) growth stages for N_con_, N_opt_ and N_opt/L_; N_80%opt/L-1_, 80% of optimized urea amended with Limus (120 kg N ha^−1^) applied only once at V0 or V3 stage. The additional treatment was N_opt/L-1_ at SZ, which was optimized urea amended with Limus (150 kg N ha^−1^) applied only once at V0 or V3, or at QZ, N_80%opt/L_ which was optimized urea amended with Limus (120 kg N ha^−1^) applied half at V3 and half at V12. The amount of optimized urea N was based on previous research on N fertilizer recommendations^38^,^39^. V3 and V12 were two key stages of maize for N topdressing. The maize variety *Xianyu 335* was used at SZ at sowing densities of 60,000 and 70,000 seeds ha^−1^ in the first and second years, respectively. The maize variety at QZ was *Zhengdan 958* sown at 90,000 seeds ha^−1^, based on local practice. Maize crop management (e.g. applications of pesticide and herbicide) was conducted according to traditional local practices at each site. Before the experiment, the winter wheat and summer maize rotation had been planted for at least one year without fertilizer inputs. No irrigation water was supplied for maize production at each site.

### NH_3_ loss measurements

A calibrated Dräger-Tube Method (DTM) was adopted to measure NH_3_ loss, which is a dynamic chamber technique adjusted to actual meteorological conditions (e.g., temperature, wind speed) by an empirical calibration equation^8^. The calibrated DTM has been shown to be well-suited for NH_3_ measurements in multi-plot trials, and has been widely used around the world^40^,^41^,^42^. Briefly, four chambers (total surface area approx. 400 cm^2^) were gently pressed about 2 cm into the soil, and connected to a Teflon tube, a Dräger NH_3_ indicator tube, and a hand pump to detect the NH_3_ concentration. The primary NH_3_ flux was converted into the ambient NH_3_ flux using a calibration equation that included outside temperature and wind speed (at 0.2 m and 2 m height). The NH_3_ indicator tubes and pump were supplied by Drägerwerk AG, Lübeck, Germany. More details of the calibrated DTM method are presented in^40^,^43^, where the comparison of the DTM with NH_3_ volatilization observed from micrometeorological measurements showed very good agreement, especially for surface applied and evenly distributed N fertilizers.

### Sampling and laboratory procedures

The maize plants were harvested manually and aboveground crop residues, except stubble, were removed from each plot at maturity during the two maize cropping seasons. The plant samples (including grains, cobs and straws) from six maize plants per plot were oven-dried at 65 °C to a constant weight. For determination of above-ground N uptake, plant samples were digested by H_2_SO_4_-H_2_O_2_ solution and N concentrations in grain and straw analyzed by the micro-Kjeldahl method.

Soils were sampled with an auger to 1 m before planting and after harvest, taking five cores per plot in 20-cm increments (i.e. 0–20, 20–40, 40–60, 60–80 and 80–100 cm). The fresh soil samples were extracted with a 1:10 ratio of soil to 0.01 mol L^−1^ CaCl_2_ and analyzed for NO_3_^−^-N and NH_4_^+^-N by an AA3 continuous flow analyzer (Bran + Luebbe GmbH, Norderstedt, Germany).

### Calculations and Statistical analysis

The RE_N_ was calculated using the following equation: RE_N_ = (crop N uptake in applied N plot-crop N uptake in N_0_ plot)/applied fertilizer N × 100 and indicates the percentage of fertilizer N recovered in aboveground plant biomass^44^.

Other components of N balances (e.g., Apparent N mineralization, N surplus, N loss) were estimated using the following equations^37^,^45^,^46^:


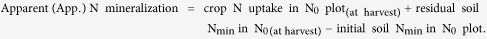



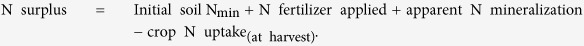



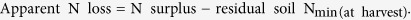



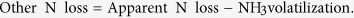


where the initial soil N_min_ and residual soil N_min_ were the mineral N content within the top 1 m of soil before planting and after harvest, respectively.

One-way analysis of variance was performed on NH_3_ loss, grain yield, RE_N_ and other related parameters using the SPASS 17.0 statistical software. Significant differences among means were identified using Duncan’s test at *P* < 0.05.

## Additional Information

**How to cite this article**: Li, Q. *et al*. A new urease-inhibiting formulation decreases ammonia volatilization and improves maize nitrogen utilization in North China Plain. *Sci. Rep.* **7**, 43853; doi: 10.1038/srep43853 (2017).

**Publisher's note:** Springer Nature remains neutral with regard to jurisdictional claims in published maps and institutional affiliations.

## Figures and Tables

**Table 1:** Experimental treatments at Shangzhuang (SZ) and Quzhou (QZ) (unit: kg N ha^−1^).

Site	Treatment	1^st^ N applied (V0 or V3 stage)	2^nd^ N applied(V12 stage)	Total N input
SZ	N_0_	0	0	0
	N_con_	150	150	300
	N_opt_	60	90	150
	N_opt/L_	60	90	150
	N_opt/L-1_	150	0	150
	N_80%opt/L-1_	120	0	120
QZ	N_0_	0	0	0
	N_con_	135	135	270
	N_opt_	75	75	150
	N_opt/L_	75	75	150
	N_80%opt/L_	60	60	120
	N_80%opt/L-1_	120	0	120

**Table 2:** Cumulative NH_3_ losses (unit: kg N ha^−1^) following urea (±Limus) application to the different treatments during the two years of maize production at Shangzhuang (SZ) (2011 and 2012) and Quzhou (QZ) (2012 and 2013).

Site	Treatment	1^st^ Year			2^nd^ Year		
		V0/V3	V12	Total loss	V3	V12	Total loss
SZ	N_con_	0	11c^*^	11c	31c	6c	37c
	N_opt_	0	9b	9b	23b	5b	28b
	N_opt/L_	0	2a	2a	0a	0a	0a
	N_opt/L-1_	0	n.d.	0a	1a	n.d.	1a
	N_80%opt/L-1_	0	n.d.	0a	1a	n.d.	1a
QZ	N_con_	24c	31c	55c	50c	59c	109d
	N_opt_	18b	24b	42b	40b	46b	86c
	N_opt/L_	3a	1a	4a	12a	17a	29b
	N_80%opt/L_	3a	1a	4a	13a	17a	30b
	N_80%opt/L-1_	5a	n.d.	5a	17a	n.d.	17a

Note that values without the same letters within the same column at each site are significantly different (*P* < 0.05, Duncan’s test). n.d. means not detectable.

**Table 3:** Nitrogen balances in the two maize seasons at SZ and QZ (unit: kg N ha^−1^).

Year	Item	N_0_	N_con_	N_opt_	SZ site N_opt/L_	N_opt/L-1_	N_80%opt/L-1_	N_0_	N_con_	N_opt_	QZ site N_opt/L_	N_80%opt/L_	N_80%opt/L-1_
**1**^**st**^ **Year**	**N input**	**81**	**381**	**231**	**231**	**231**	**201**	**85**	**355**	**235**	**235**	**205**	**205**
	Initial soil N_min_	68	68	68	68	68	68	35	35	35	35	35	35
	Applied N	0	300	150	150	150	120	0	270	150	150	120	120
	App. N mineralization	13	13	13	13	13	13	50	50	50	50	50	50
	**N output** (**N uptake**)	**51**	**141**	**129**	**118**	**128**	**103**	**64**	**173**	**144**	**169**	**155**	**136**
	**N surplus**	**30**	**240**	**102**	**113**	**103**	**98**	**21**	**182**	**91**	**66**	**50**	**69**
	Residual soil N_min_	30	212	72	110	86	84	21	105	37	48	39	40
	Apparent N loss	0	28	30	3	17	14	0	77	54	18	11	29
	NH_3_ volatilization	0	10	9	3	0	0	0	55	42	4	4	5
	Other N loss	0	18	21	0	17	14	0	22	12	14	7	24
**2**^**nd**^ **Year**	**N input**	**114**	**572**	**311**	**332**	**321**	**280**	**123**	**454**	**306**	**312**	**274**	**276**
	Initial soil N_min_	62	220	109	130	119	108	59	120	92	98	90	92
	Applied N	0	300	150	150	150	120	0	270	150	150	120	120
	App. N mineralization	52	52	52	52	52	52	64	64	64	64	64	64
	**N output** (**N uptake**)	**96**	**152**	**142**	**148**	**153**	**142**	**102**	**185**	**177**	**193**	**171**	**161**
	**N surplus**	**18**	**420**	**169**	**184**	**168**	**138**	**21**	**269**	**129**	**119**	**103**	**115**
	Residual soil N_min_	18	337	110	162	122	88	21	110	32	49	45	59
	Apparent N loss	0	83	59	22	46	50	0	159	97	70	58	56
	NH_3_ volatilization	0	37	28	0	1	1	0	108	85	30	31	17
	Other N loss	0	46	31	22	45	49	0	51	12	40	27	39

Initial soil N_min_ and residual soil N_min_ were the mineral N content within the top 1 meter of soil before planting and after harvest, respectively. N surplus equals N input minus crop N uptake.
